# Urbanization erodes niche segregation in Darwin's finches

**DOI:** 10.1111/eva.12721

**Published:** 2018-12-18

**Authors:** Luis F. De León, Diana M. T. Sharpe, Kiyoko M. Gotanda, Joost A. M. Raeymaekers, Jaime A. Chaves, Andrew P. Hendry, Jeffrey Podos

**Affiliations:** ^1^ Department of Biology University of Massachusetts Boston Boston Massachusetts; ^2^ Centro de Biodiversidad y Descubrimiento de Drogas Instituto de Investigaciones Científicas y Servicios de Alta Tecnología (INDICASAT‐AIP) Panamá City Panamá; ^3^ Redpath Museum, Department of Biology McGill University Montréal Quebec Canada; ^4^ Department of Zoology University of Cambridge Cambridge UK; ^5^ Centre for Biodiversity Dynamics, Department of Biology Norwegian University of Science and Technology Trondheim Norway; ^6^ Laboratory of Biodiversity and Evolutionary Genomics University of Leuven Leuven Belgium; ^7^ Colegio de Ciencias Biológicas y Ambientales Universidad San Francisco de Quito, Diego de Robles y Pampite Quito Ecuador; ^8^ Galápagos Science Center Puerto Baquerizo Moreno Galápagos Ecuador; ^9^ Department of Biology University of Massachusetts Amherst Amherst Massachusetts

**Keywords:** adaptive divergence, Galápagos, human disturbances, maladaptation, niche partitioning, resource distribution, urban ecology, urbanization

## Abstract

Urbanization is influencing patterns of biological evolution in ways that are only beginning to be explored. One potential effect of urbanization is in modifying ecological resource distributions that underlie niche differences and that thus promote and maintain species diversification. Few studies have assessed such modifications, or their potential evolutionary consequences, in the context of ongoing adaptive radiation. We study this effect in Darwin's finches on the Galápagos Islands, by quantifying feeding preferences and diet niche partitioning across sites with different degrees of urbanization. We found higher finch density in urban sites and that feeding preferences and diets at urban sites skew heavily toward human food items. Furthermore, we show that finches at urban sites appear to be accustomed to the presence of people, compared with birds at sites with few people. In addition, we found that human behavior via the tendency to feed birds at non‐urban but tourist sites is likely an important driver of finch preferences for human foods. Site differences in diet and feeding behavior have resulted in larger niche breadth within finch species and wider niche overlap between species at the urban sites. Both factors effectively minimize niche differences that would otherwise facilitate interspecies coexistence. These findings suggest that both human behavior and ongoing urbanization in Galápagos are starting to erode ecological differences that promote and maintain adaptive radiation in Darwin's finches. Smoothing of adaptive landscapes underlying diversification represents a potentially important yet underappreciated consequence of urbanization. Overall, our findings accentuate the fragility of the initial stages of adaptive radiation in Darwin's finches and raise concerns about the fate of the Galápagos ecosystems in the face of increasing urbanization.

## 1 INTRODUCTION

One of the hallmarks of adaptive radiation is niche segregation, whereby closely related populations or species evolve to specialize on distinct ecological resources (Grant, 1999; Lack, 1947; Schluter, 2000; Simpson, 1953). Niche segregation is thought to emerge principally from competition for shared ecological resources (Gause, 1934; Hardin, 1960; Macarthur & Levins, 1967; Roughgarden, 1976; Schoener, 1968) and is determined jointly by the availability of ecological resources and the ability of consumer populations to exploit those resources. Accordingly, variation in resource distributions can facilitate niche segregation between populations or species in a given environment (De León, Podos, Gardezi, Herrel, & Hendry, 2014; Levine & HilleRisLambers, 2009; Pianka, 1973; Schoener, 1974; Tilman, 1982). Niche segregation can also be favored by additional factors, including behavioral flexibility or phenotypic plasticity, whereby individuals explore novel resources within shared environments (Boogert, Monceau, & Lefebvre, 2010; Ducatez, Clavel, & Lefebvre, 2015; Inouye, 1978; Nicolakakis, Sol, & Lefebvre, 2003; Sol, González‐Lagos, Moreira, Maspons, & Lapiedra, 2014; Wright, Eberhard, Hobson, Avery, & Russello, 2010) or by genetically based phenotypic variability, whereby individuals with different trait values exploit and diverge into resources to which they are best adapted (Bolnick & Paull, 2009; Bolnick, Svanbäck, Araújo, & Persson, 2007; De León, Rolshausen, Bermingham, Podos, & Hendry, 2012).

The process of divergence via niche segregation can be conceptualized as the splitting of populations along a “rugged” adaptive landscape—a surface relating the mean fitness of populations or species to mean trait values, with “ruggedness” arising from distinct alternative fitness peaks that correspond to different ecological resources (Simpson, 1953; Svensson & Calsbeek, 2012). As such, alterations to resource availability and resource distributions are viewed as affecting the shape of adaptive landscapes underlying diversification (De León et al., 2011; Hendry et al., 2006). Alterations to adaptive landscapes can be particularly drastic in the case of human disturbances such as urbanization, where large swathes of natural environments—and the resources they contain—are altered by many factors, including infrastructure development, the introduction of exotic species, and human food availability (Alberti, 2015; Aronson et al., 2014; Gaston, 2010; Gotanda, Hendry, & Svensson, 2017; McKinney, 2002, 2006 ; Penick, Savage, & Dunn, 2015). However, despite the rapid increase in urbanization worldwide (Grimm et al., 2008; Seto, Sánchez‐Rodríguez, & Fragkias, 2010; Wigginton, Fahrenkamp‐Uppenbrink, Wible, & Malakoff, 2016), and accumulating evidence that some species can adapt accordingly (Donihue & Lambert, 2014; Johnson & Munshi‐South, 2017; Kettlewell, 1955; Littleford‐Colquhoun, Clemente, Whiting, Ortiz‐Barrientos, & Frère, 2017; Lowry, Lill, & Wong, 2013; Slabbekoorn & Peet, 2003; Winchell, Reynolds, Prado‐Irwin, Puente‐Rolón, & Revell, 2016), the exploitation of human‐introduced ecological resources has not yet been linked to the alteration of specific ecological niches or adaptive landscapes that drive diversification in nature. Here, we explore such links in Darwin's ground finches (*Geospiza* spp.) across sites with different degrees of urbanization on Santa Cruz Island, Galápagos, Ecuador. Specifically, we ask the following: (a) Has the availability of novel human foods in urban areas altered finch diets?; and, if so, (b) Do finches in urban environments prefer human foods over natural foods?; (c) Do finches in urban areas respond differently to the presence of people?; and (d) What are the consequences of finches' use of human foods for the persistence of ecological differences underlying the finch adaptive radiation?

In the adaptive radiation of Darwin's finches, beak morphology has diversified as a consequence of adaptation to different ecological resources. For instance, in the ground finches, divergent beak sizes and shapes are considered adaptations to exploit different seed types (Supporting Information Figure S1), presumably corresponding to different peaks on their adaptive landscape (Abbott, Abbott, & Grant, 1977; Bowman, 1961; Grant & Grant, 2008; Lack, 1947; Schluter & Grant, 1984). Specifically, the small, medium, and large ground finches (*Geospiza fuliginosa, G. fortis,* and *G. magnirostris*) feed on small/soft, medium, and large/hard seeds, respectively, and accordingly have evolved small, medium, and large beaks. The closely related cactus finch (*Geospiza scandens)* specializes on the nectar, pollen, and seeds of *Opuntia* cacti and has evolved an elongated beak (Supporting Information Figure S1). Resource partitioning has also promoted intra‐specific adaptive divergence within the medium ground finch, where two beak‐size morphs on Santa Cruz Island have diverged significantly in ecological (De León et al., 2014, 2012), morphological (Hendry et al., 2006; Hendry, Huber, León, Herrel, & Podos, 2009; Huber, Leon, Hendry, Bermingham, & Podos, 2007), and genetic attributes (Chaves et al., 2016; De León, Bermingham, Podos, & Hendry, 2010; Huber et al., 2007). Resource partitioning has also been associated with morphological divergence between highland and lowland populations of the small ground finches on Santa Cruz Island (Kleindorfer, Chapman, Winkler, & Sulloway, 2006; Sulloway & Kleindorfer, 2013). Studies on the continuum of intra‐ to inter‐specific divergence in the ground finches can help reveal the processes underlying adaptive divergence and how it might be influenced by urbanization.

Previous work on the medium ground finch suggests that divergence of the morphs has recently diminished at sites adjacent to a human settlement (Hendry et al., 2006). It has been hypothesized that the apparent recent fusion of beak‐size distributions of the morphs, from bimodal to unimodal, was due to the introduction and ready availability of human foods, which might be flattening the adaptive landscape and thereby reducing selection against intermediate forms (De León et al., 2011; Hendry et al., 2006). A critical test of this hypothesis would involve asking whether niche segregation and feeding preferences actually differ between urban and non‐urban contexts. In the present paper, we offer such a test, by conducting feeding observations and field experiments on coexisting ground finch species at sites that span different degrees of urbanization.

## 2 METHODS

### 2.1 Field sites

**Figure 1:**
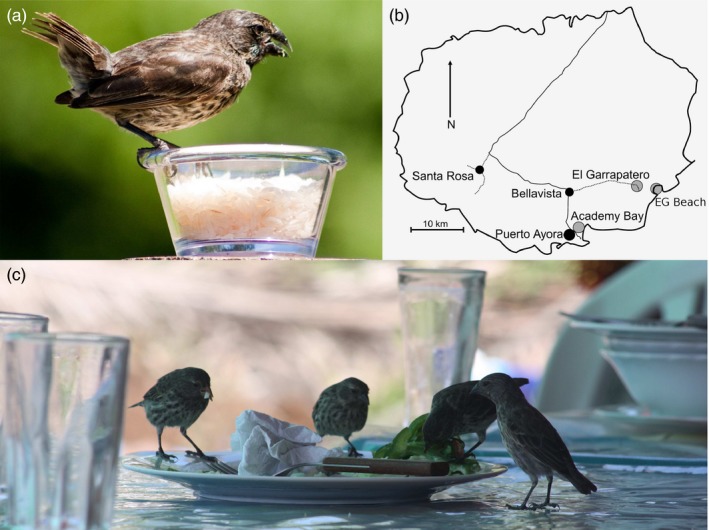
Finches feeding on human foods at urban sites. Panels show a female medium ground finch eating dry rice from a feeder (a). Study sites on Santa Cruz Island, Galápagos, Ecuador, with urban (black dots) sites, non‐urban sites (gray dots), and roads (lines) designed for vehicular traffic (b). Santa Rosa and Bellavista were not included in our study, but are shown here for illustrative purpose only. A group of small ground finches feeding crumbs off a plate at restaurant in Puerto Ayora (c). Photo credit: L. F. De León

Sampling and experiments took place between January and March of 2014 and 2015 at four sites on Santa Cruz Island, Galápagos, Ecuador (Figure 1). All four sites are located within the low‐elevation arid zone of the island (Wiggins & Porter, 1971) and differed in their degrees of urbanization as well as human‐associated activities (i.e., the tendency of people to feed finches; see Table 1 for details). The foraging ecology and food preferences of finches are likely to differ in transition or high‐elevation sites, and therefore, responses to urbanization may also differ in these zones.

**Table 1:** Level of urbanization and human behavior at each study site on Santa Cruz Island, Galápagos, Ecuador

Site	Urbanization level	Annual visitors	Tendency of feeding	Human contact
El Garrapatero	Non‐urban	Only scientists visit this site	No human feeding; low human density	Minimal contact with humans, except for scientists and park rangers
El Garrapatero Beach	Non‐urban tourist	38,542	Regular human feeding; low human density	Humans visit beach for the day and bring snacks/picnics as there are no shops
Academy Bay	Intermediate urban	78,555	Little human feeding, but finches feed opportunistically on food scraps, and are likely to be intentionally fed on occasions; high human density	Humans visit research center and are advised not to feed the finches, but finches are often within close proximity to large groups of people
Puerto Ayora	Urban	158,339	Regular human feeding; high human density	Humans in city generate food scraps on an hourly basis

Note

Sites are ranked according to the tendency of humans to feed finches (tendency of feeding) and the degree of interaction (human contact) with humans at each site. Data for the annual number of visitors were obtained from Dirección del Parque Nacional Galápagos & Observatorio de Turismo de Galápagos (2016).

The first site, El Garrapatero (EG; “non‐urban” site; 0°41'16.6"S 90°13'19.4"W), is located 1–2 km inland of the island's eastern coast and is about 10 km from the nearest major human settlement (Bellavista, Figure 1). Introduced plant species and human foods are rare at this site (De León et al., 2011), although human activity has increased since 2009 due to the paving of a road that provides access to the coast. On a typical day, dozens of vehicles now pass through the site. Browsing by feral goats and donkeys was historically common at EG, but eradication efforts led by the Galápagos National Park (Phillips, Wiedenfeld, & Snell, 2012) have resulted in decreased grazing disturbance. Surveys at this site were performed in an area of ~0.5 km^2^ eastward from EG road.

The second site, EG Beach (“non‐urban tourist” site; 0°41'39.9"S 90°13'15.8"W), hereafter referred to as “EG Beach,” is located on the eastern shore of the island adjacent to the first site (Figure 1). The number of visitors to this site has increased markedly due to the newly paved road, which provides direct access to the beach. Although this site supports no permanent human presence, infrastructure has expanded over the past 6 years to include a gravel parking lot, cobblestone paths, a ranger outpost, and an overnight camping ground. Typically, 5–20 tourists a day (although sometimes many more) visit the beach to swim, kayak, picnic, and observe wildlife. We included this site to help disentangle two urban factors: the large‐scale alteration of habitats (absent here) versus the occasional to regular presence of humans themselves (present here).

The third site, Academy Bay (AB; “intermediate urban” site; 0°44'31.6"S 90°18'15.3"W), is situated on the south coast of the island and is contiguous with the town of *Puerto Ayora* (PA). Human influences at AB include wide cobblestone and dirt paths, a high abundance of exotic plant species, and the presence of human foods associated mainly with concessions for tourists visiting the Charles Darwin Research Station (CDRS), as well as a few local residents and dorms. Finches at this site are regularly observed consuming human foods (Figure 1) and drinking freshwater from tortoise pens and broken pipes (De León et al., 2011). Surveys at this site were performed in an area of ~0.5 km^2^ encompassing trails from the public entrance of the Galápagos National Park eastward to a cliff behind the animal facilities of the CDRS and did not include the beach areas bordering the CDRS.

The fourth site, PA (“urban” site; 0°44'34.8"S 90°18'43.4"W), is the largest human settlement on Galápagos with over 12,000 inhabitants (Instituto Nacional de Estadística y Censos). *Puerto Ayora* also receives many more tourists than other islands in the Archipelago, with ~218,365, and 241,800 recorded visitors in 2016 and 2017, respectively (Dirección del Parque Nacional Galápagos & Observatorio de Turismo de Galápagos, 2016, 2017). At this site, we have seen finches feeding on a wide variety of introduced plant species and human foods (Figure 1), including bread, potato chips, ice‐cream cones, rice, and beans (De León et al., 2011). Surveys at this site were performed in an area of ~1.0 km^2^ encompassing the fire station, the farmer's market, the cemetery, the public dock, and the entrance to the trail to Tortuga Bay.

Given the complex ways in which finches could interact with humans on Santa Cruz Island (Table 1), we cannot consider our four sampling sites as representing a simple urbanization gradient. We rather consider them as four discrete sites that vary independently in both the degree of urbanization (as given by human infrastructure and population density) and the potential for human interaction with finches (a function of both the number and behavior of tourists).

Regarding human interaction with finches, reports from the Galapagos National Park indicate that the number of visitors to the Galápagos is high during the entire year, with two inter‐annual peaks: the first between July and September, and the second in March (Dirección del Parque Nacional Galápagos & Observatorio de Turismo de Galápagos, 2016, 2017 ). Thus, our sampling period (January–March) occurred just before the onset of the second largest peak in the number of visitors. Although we did not quantify food availability in the current study, finches in urban areas are likely to enjoy a surplus of human foods throughout the year. However, we do not expect that this supply was any higher during our sampling period. Further studies will be necessary, however, to better understand how finch preferences for human foods might respond to temporal variation in the availability of both natural and human foods.

### 2.2 Feeding observations

Our first goal was to quantify the diets at our study sites of the four *Geospiza* species (*G. fortis*,* G. fuliginosa*,* G. scandens,* and *G. magnirostris*). Toward this end, we employed a point observation method (De León et al., 2014, 2011, 2012 ). Briefly, during morning or afternoon hours, we walked along predetermined transects and used binoculars to identify birds (to the species level) and, if possible, the food items on which they were feeding. At three of our sites, we surveyed along a total of 74 transects covering a linear distance of 30.74 km: EG (*n* = 22, mean length = 436.80 m), AB (*n* = 22, 476.75 m), and PA (*n* = 30, 353.84 m). No feeding observations or estimations of bird density were performed at EG Beach because this site is represented by an open sandy beach with little natural vegetation. Transect courses were determined randomly at the beginning of each walk, but they were limited to a series of preexisting trails that facilitated access to sites with dense vegetation (EG and AB). In the town of PA, transects were determined by following both large and small streets through the middle and around the town, including surrounding neighborhoods (Miraflores and El Edén). Observations in this area included finches found on the streets, sidewalks, and restaurant areas, as well as on the natural vegetation of parks, gardens, and roadsides.

Food items included specific plant species and plant parts (i.e., seeds, fruits, leaves, or flowers) as well as different types of human foods. We also recorded the category “ground,” when birds were feeding on the ground, but the exact food items could not be identified owing to their small size. After a feeding event was recorded, we moved immediately onto the next individual to avoid pseudoreplication. Our data therefore represent counts of discrete observations of individual birds feeding on particular food items (De León et al., 2014, 2012 ). Finally, we generated rarefaction curves to visualize how the cumulative numbers of food items observed varied in relation to our sampling efforts at each site.

### 2.3 Bird density

Our second goal was to estimate variation in bird density across sites. For this task, we used bird count data for our focal species from our feeding observation transects, given that we recorded all birds within 30 m at each side of the observer, whether they were feeding or not. We then used these values to estimate the number of individuals per unit area (Emlen, 1977). Two factors could have affected our estimates of bird density. The first is bird detectability at sites with dense vegetation, such as EG and AB, in contrast to the more open urban site (PA). And second, combining feeding observations and bird count along the same transect might not be as accurate as surveys dedicated to bird counts alone. However, our main goal here was to estimate *relative* differences in bird abundance between urban and non‐urban environments, rather than providing a precise value of bird density at each site. In addition, to reduce autocorrelation effects and to improve detectability we recorded only one feeding event per individual (see above), and only within 30 m of the observer. These types of observations were also facilitated by the fact that Darwin's finches are tame and can be easily observed at short distances with little interference (De León et al., 2014; Grant, 1999).

### 2.4 Finch response to food cues

To test whether and how finches across our study sites respond to the presence of people, we developed a “finch–human interaction” experiment. We recorded finch responses to two different human stimuli: a visual stimulus (an experimenter standing or sitting still and quietly in an open space) and an audiovisual stimulus (an experimenter standing or sitting still and noisily rustling a bag of potato chips, generating a “crinkle” sound typically associated with packaged human foods). Including this second stimulus was inspired by observations of finches approaching humans opening and/or handling packaged foods. The stimuli were presented sequentially and in the same spot, with the visual stimulus first (5 min) and the audiovisual stimulus second (five more minutes). During stimulus presentation, we recorded the number, species, and sex of all birds that approached within 1.5 m of the experimenter, including birds that perched above the experimenter. Presentation locations within our sites were selected haphazardly and were conducted at least 100 m apart from each other during a given day. Data for this experiment were collected at all four sites: EG (non‐urban, *n* = 22), EG beach (non‐urban tourist, *n* = 37), AB (intermediate urban, *n* = 14), and PA (urban, *n* = 30).

### 2.5 Feeding preference experiment

To quantify finch feeding preferences, and whether and how they varied with the degree of urbanization, we performed replicate “cafeteria” experiments, in which finches were presented with a choice of human and native food items. We constructed cardboard feeding trays (30 cm × 30 cm) with nine sections (3 × 3 grid pattern) into which food could be placed. A rock was placed in the center section of each tray as an anchor. Each tray was stocked with 2.5 g of each of six natural or human foods commonly observed previously (De León et al., 2014, 2011, 2012 ). To control for any potential biases associated with the location of food on the tray, food items were placed randomly in six of the eight available sections of the tray. Human food items included uncooked white rice, potato chips, and coconut cookies, the latter two of which we crumbled into small pieces to mimic the size and shape commonly seen in urban sites. For natural native foods, we included fruits from *Cryptocarpus pyriformis*,* Tournefortia psilostachya,* and *Scutia spicata*. We choose these three plant species because they occur commonly at each of our sites and are eaten frequently by all ground finches, regardless of their beak size (De León et al., 2014). Furthermore, they all produce small and soft seeds, and therefore are comparable to human foods in terms hardness, and are unlikely to impose functional constraints on finch feeding. We did not include additional soft food items such as insect larvae in our experiments because their most important contribution to finch diet is limited to the onset of the rainy season (De León et al., 2014); thus, they represent a less stable food resource for finches when compared with other natural and human foods items. The tray was placed on the ground, and observers moved at least 10 m away or concealed themselves at least 5 m away to record feeding activity. We did not present empty trays in our trials because we were interested in finch preference for different types of foods rather than finches' reactions to the presence of food in general. Trial sites were selected haphazardly, and no trials were conducted within 100 m of each other during the same day. Trays were left out for a maximum of 20 min if no finch approached. If a finch approached and fed from the tray, a timer was started and observations recorded for 10 min from the first finch feeding. We recorded the total number of finches that approached, perched on, and/or fed at the tray, and their species identity. At the end of each trial, trays were collected and the food that remained in each section re‐weighed. We performed trials at all four sites: EG (*n* = 34), EG beach (*n* = 40), AB (*n* = 31), and PA (*n* = 46). Overall, our experiments were not designed to disentangle the mechanisms underlying natural feeding preferences in Darwin's finches, but rather to test whether or not Darwin finches show preferences for human foods over natural foods in both urban environments and non‐urban environments.

### 2.6 Data analysis

To characterize variation in finch diets across sites, we performed correspondence analysis (CA) on our feeding observation data. CA is a multivariate descriptive analysis based on matrices of frequency data (Benzécri, 1973). We here used CA to visualize and determine the contribution of each food item to the total diet of each species at each site. We then used cosine‐squared (Cos^2^) correlations (R package FactorMineR; Lê, Josse, & Husson, 2008) to test for associations among food items and finch diets. To test for variation in bird density across sites, we performed a Kruskal–Wallis *H* test, followed by a post hoc Dunn test for multiple comparisons.

To assess among‐site variation in finch response to humans, we ran two tests. First, we tallied the number of birds approaching human experimenters in the response trials and performed a Kruskal–Wallis test on these tallies. Second, for each site we calculated the proportion of trials in which at least one finch approached the stimulus, relative to the total number of trials performed (proportion positive response) and performed a chi‐squared test on these proportions.

To analyze feeding preferences from our cafeteria experiment, we constructed a nested linear model to test for variation in the amount of food eaten (in grams) across sites, food category (human vs. natural), food item nested within food category (rice vs. chips vs. cookies for human foods and *C. pyriformis* vs. *T. psilostachya* vs. *S. spicata* for natural foods), and the two‐way interactions (site × food category and site × food item). The number of birds that approached trays in the cafeteria experiments provided us with a third metric of response, beyond the two noted in the previous paragraph.

To test for site‐specific variation in dietary niche partitioning, we estimated at each site two commonly considered niche parameters: niche width within each species, and niche overlap between each species pair. Analyses were performed across all ground finches together (*n* = 1,063) and for the two ground finch species that had the highest sample sizes (*G. fortis* [*n* = 571] and *G. fuliginosa* [*n* = 349]) (Table 2). To estimate niche width, we calculated Shannon's (Colwell & Futuyma, 1971) and Levin's (Levins, 1968) niche width indices as implemented in the R package spaa v.0.2.2 (Zhang, 2016). To estimate niche overlap, we calculated Pianka's (1973) niche overlap indices as implemented in the R package EcoSimR (Gotelli & Ellison, 2013). These indices range from zero to one, with zero indicating no overlap in food items across species and one indicating complete overlap. Following De León et al. (2014), we next used EcoSimR to generate null models of expected niche overlap under different randomization algorithms (Gotelli & Entsminger, 2009). With these models, we tested whether observed niche overlap between species differed significantly from random expectations. We generated 1,000 permutations under the RA3 algorithm (Lawlor, 1980). The RA3 algorithm is recommended for niche overlap estimates because it randomizes each species' prey items, while maintaining its overall niche breadth, thus generating a random utilization matrix with similar dimensions as the observed matrix (Gotelli & Entsminger, 2009; Lawlor, 1980). All data analysis and graphing were performed in R version 3.3.0 (R Core Team, 2013). Research permits for this work were obtained from the Ministry of Environment of Ecuador and the Galápagos National Park (Permits No. PC‐29‐14 and PC‐25‐15). This study was also conducted following the guidelines of the animal care protocol of the University of Massachusetts, Amherst.

**Table 2:** Number of feeding observations across three sites and 11 passerine bird species on Santa Cruz Island, Galápagos, Ecuador

Species	Common name	El Garrapatero		Academy Bay		Puerto Ayora		Total
		Human	Natural	Human	Natural	Human	Natural	
*Geospiza fortis*	Medium ground finch	3	102	9	173	227	57	571
*Geospiza fuliginosa*	Small ground finch		31	11	133	152	22	349
*Geospiza magnirostris*	Large ground finch				3	3	5	11
*Geospiza scandens*	Cactus finch		6	4	30	7	8	55
*Geospiza* spp.	Ground finch		16	6	24	30	1	77
*Geospiza parvula*	Small tree finch		2		7	10	1	20
*Platyspiza crassirostris*	Vegetarian finch		5	3	72	11	15	106
*Crotophaga ani*	Smooth‐billed ani					1	1	2
*Setophaga petechia*	Yellow warbler			1	8		3	12
*Mimus parvulus*	Galápagos mockingbird		1		5			6
*Myiarchus magnirostris*	Galápagos flycatcher				2		2	4
Total		3	163	34	457	441	115	1,213

Note

Observations of other species are included here for reference, but only data from ground finches (*Geospiza* spp.) were included in our statistical analyses. When identification to species level was not possible, we included a *Geospiza* spp. category. For each observation, we reported each food item's origin, natural versus human.

## 3 RESULTS

### 3.1 Feeding observations and bird density

During the 2 years of our study, we collected a total of 1,213 feeding observations: 166 at EG, 491 at AB, and 556 at PA (Table 1; Supporting Information Table S1). The total number of different food items we observed finches eating corresponded roughly to degrees of urbanization, from 13 at EG to 31 at AB to 36 at PA (Table 1 and Supporting Information Table S1). The greater diversity of food types at the latter sites is due mostly to the presence at these sites of a variety (11 items) of human foods (Table 1; Supporting Information Table S1). By contrast, the diversity of native plant species consumed was similar across sites, consistent with findings from our previous 5‐year survey at EG and AB (De León et al., 2014). Although the number of feeding observations varied across sites, rarefaction curves showed evidence for saturation at a relatively low number of observations (~150) for each site (Supporting Information Figure S2), suggesting that our sampling effort was sufficient to estimate dietary niches in ground finches. This was also consistent with a previous 5‐year study of feeding preference at two of the same sites (EG and AB) (De León et al., 2014).

For our transect surveys, we observed a total of 3,343 individuals (both feeding and non‐feeding) during 75.1 hr (Supporting Information Table S2). The density of ground finches varied significantly across sites, mean ± *SE*: 761.37 ± 81.55 bird/km^2^ at EG, 923.69 ± 75.33 bird/km^2^ at AB and 1,019.17 ± 104.65 bird/km^2^ at PA; Kruskal–Wallis test: *χ* ^2^_(2)_ = 8.23, *p* = 0.016, with post hoc tests confirming a significant difference between the most‐urbanized sites (PA) and both the least‐urbanized site (EG, *p* = 0.003) and the intermediate‐urbanized site (AB, *p* = 0.027).

**Figure 2:**
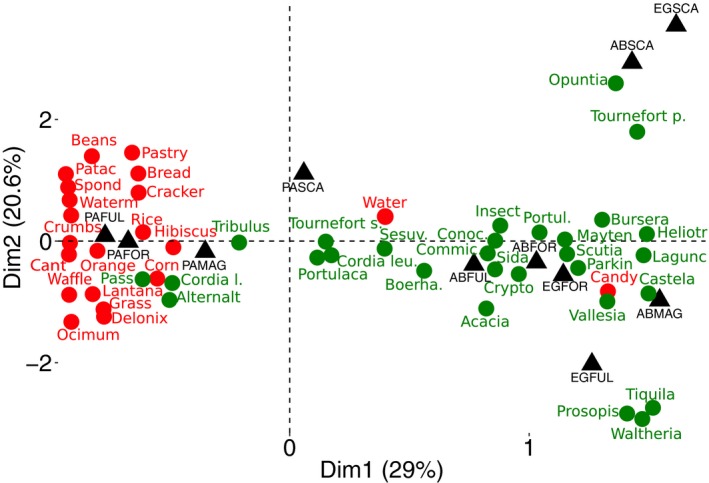
Diet of ground finches across sites with different degrees of urbanization on Santa Cruz Island, Galápagos, Ecuador. The graph represents correspondence analysis (CA) based on feeding observation data. Colors represent natural (green) and human (red) food items. Black labels represent species and site centroid combinations: El Garrapatero (EG), Academy Bay (AB), Puerto Ayora (PA), *Geospiza fortis* (FOR), *Geospiza fuliginosa* (FUL), *Geospiza magnirostris* (MAG), and *Geospiza scandens* (SCA). Food items labels and points were slightly offset to facilitate readability. In this graph, the position of each species/site combination (filled triangles) corresponds to the food items favored in its diet. Finches at the urban site, PA, cluster near the human food items

Correspondence analyses provided a further illustration of how finches consumed human food items with increasing urbanization (Figure 2, note how species at the urban site, PA, cluster near the human food items, shown as red filled circles). Some human foods contributed disproportionately to the finch diet at urban sites, but almost never at non‐urban sites (Figure 2; Supporting Information Table S1). At PA in particular, finches were observed feeding almost exclusively (78.6% of all observations) on human foods, including crackers (3.7%), rice (5.7%), bread (6.6%), and unidentified crumbs (46%), as well as introduced garden species such as *Hibiscus* sp. (1.6%), and *Delonix regia* (Flamboyant; 10.5%). At AB, finches often fed on native plant species such as *Boerhavia caribaea*,* S. spicata*,* Cryptocarpus pyriformis*, and* Cordia lutea,* but they also were seen feeding on some human food items such as bread and crumbs (Supporting Information Table S1). Finches at this site were also seen drinking freshwater from broken pipes. In contrast, at the non‐urban site (EG) finches were seen feeding almost exclusively on native species such as *S. spicata*,* C. pyriformis*,* Cordia leucophlyctis*, and *T. psilostachya* (Figure 2, Supporting Information Table S1). The only exceptions were three birds on the side of the road to the EG, which were seen pecking at a candy wrapper and crumbs left by tourists (Figure 2, Supporting Information Table S1).

### 3.2 Finch response to food cues

**Figure 3:**
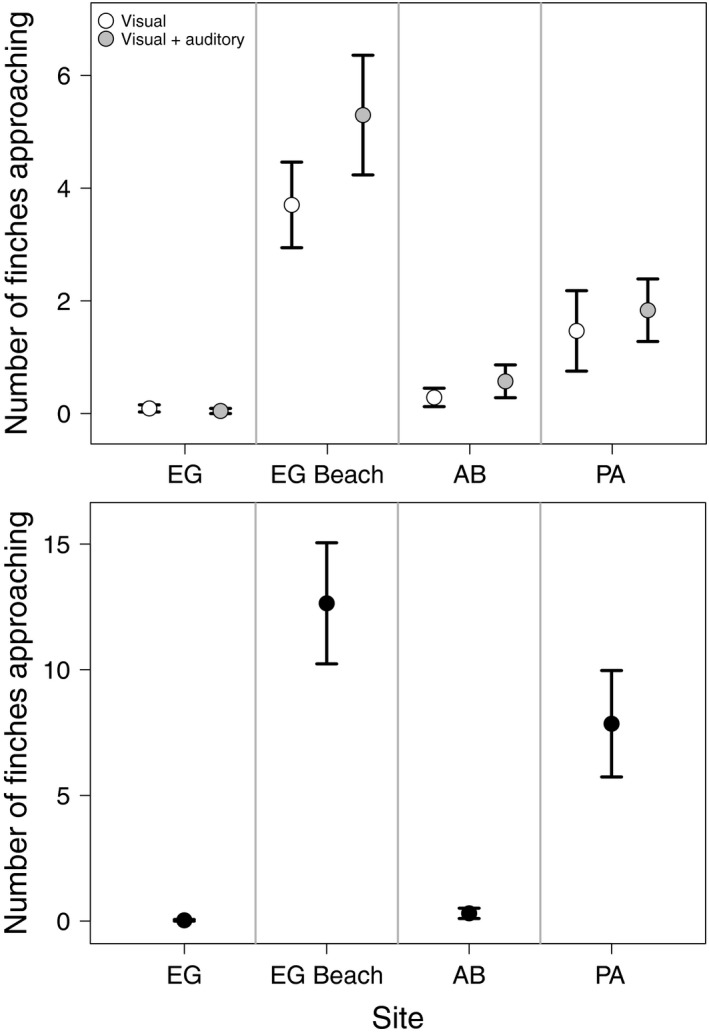
Finch response to humans across sites with different degrees of an urbanization and human behavior on Santa Cruz Island, Galápagos, Ecuador. The data represent the number of finches (mean ± *SD*) that approached the human experimenter (top panel, food preference tests) and the food tray (bottom panel, cafeteria experiment) at the four study sites. Site labels are El Garrapatero (EG), El Garrapatero Beach (EG Beach), Academy Bay (AB), and Puerto Ayora (PA)

Our finch–human interaction trials revealed significant variation across sites in finches' response to the presence of people, Figure 3; Kruskal–Wallis test: *χ* ^2^_(3)_ = 60.68, *p* < 0.001. Few finches approached the experimenters at EG (non‐urban site) and AB (intermediate‐urban site), yet many finches approached the experimenter at PA (urban site), suggesting that urban birds are indeed more accustomed to the presence of humans. Interestingly, an even stronger response (approach to an experimenter) was observed at the non‐urban tourist site, EG Beach (Figure 3; Supporting Information Table S3). Within each site, we did not find statistical differences in finch response between the audiovisual the visual stimulus alone (Figure 3; *p* > 0.05 for all comparisons). However, because the order in which we presented the stimuli (visual first and then audiovisual) was fixed rather than randomized, we were unable to distinguish the contribution of either of the stimulus from effect of longer exposure to the first stimulus to finch response. Nevertheless, the overall differences among sites in finch response to the presence of humans were corroborated by the proportion of positive responses (the number of trials in which at least one finch approached the stimulus), which varied significantly across sites, *χ* ^2^_(3)_ = 56.27, *p* < 0.001, with up to 73%, 35%, and 20% at EG Beach, PA, and AB, respectively, and only 7% at EG.

### 3.3 Feeding preference experiment

**Figure 4:**
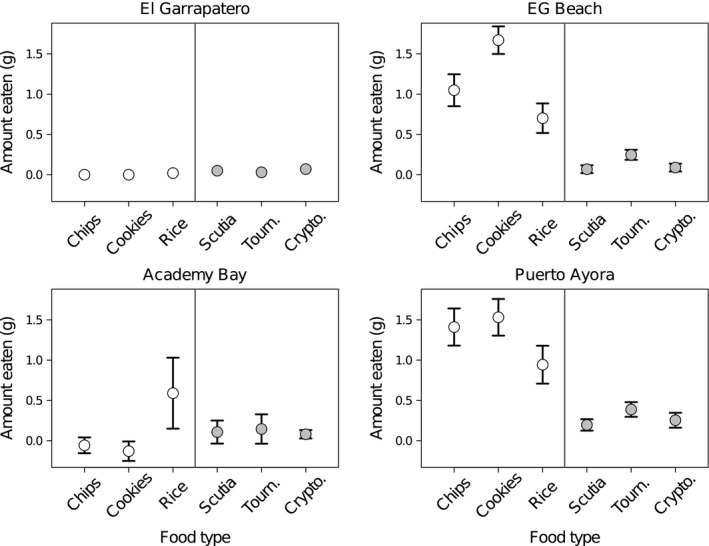
Finches prefer human food at sites where they are fed by humans. The graph shows the Mean ± *SE* of food eaten from experimental cafeteria trays at four different sites with different degrees of an urbanization and human behavior on Santa Cruz Island in the Galápagos. White indicates human foods, and gray indicates natural foods

The cafeteria experiment data revealed a striking variation in feeding preferences across sites with different degrees of urbanization (Figure 4; Supporting Information Movies S1 and Movie S2). The amount of food consumed from our experimental trays varied substantially across sites, food categories, and food items, with “site” interacting significantly with both food variables (Table 3). At EG (non‐urban site), only one individual inspected our feeding trays (Figure 3), but no food consumption was observed across any of the trials (Figure 4), even when finches were in the vicinity. At EG Beach (non‐urban, tourist site), finches preferentially consumed human foods over natural foods (Tukey's HSD: *p* < 0.001). At AB (intermediate urban site), finches showed intermediate responses to our feeding trays (Figure 4; Tukey's HSD: *p* = 0.948), with an apparent strong preference for rice over all other native and human food items (Figure 4). At PA (urban site), finches showed the strongest preference for human foods over native food items (Figure 4; Tukey's HSD: *p* < 0.001). We found that the number of finches approaching the experimental trays was lower at EG and AB than at PA and EG Beach (Figure 3). This variation was significant across sites, *χ* ^2^_(3)_ = 57.14, *p* < 0.001, and showed strong differences between EG (non‐urban) and PA (urban site) as well as between EG and EG Beach (non‐urban but tourist site) (Supporting Information Table S3).

**Table 3:** Finch feeding preferences for human versus natural foods across sites

Factors	SS	*df*	*F*	*p*
Site	2.09	3	40.723	**<0.001**
Food category	0.63	1	36.710	**<0.001**
Food type (Nested)	0.56	4	8.237	**<0.001**
Site*food category	0.95	3	18.588	**<0.001**
Site*food type	0.45	12	2.176	**0.011**
Residuals	14.26	834		

Note

Results of a nested linear model examining variation in the amount of food eaten in cafeteria experiments across sites with different degrees of urbanization on Santa Cruz Island, Galápagos, Ecuador. Values in bold represent statistically significant differences. The direction of the effects has been included in the main text.

### 3.4 Dietary niche partitioning

**Figure 5:**
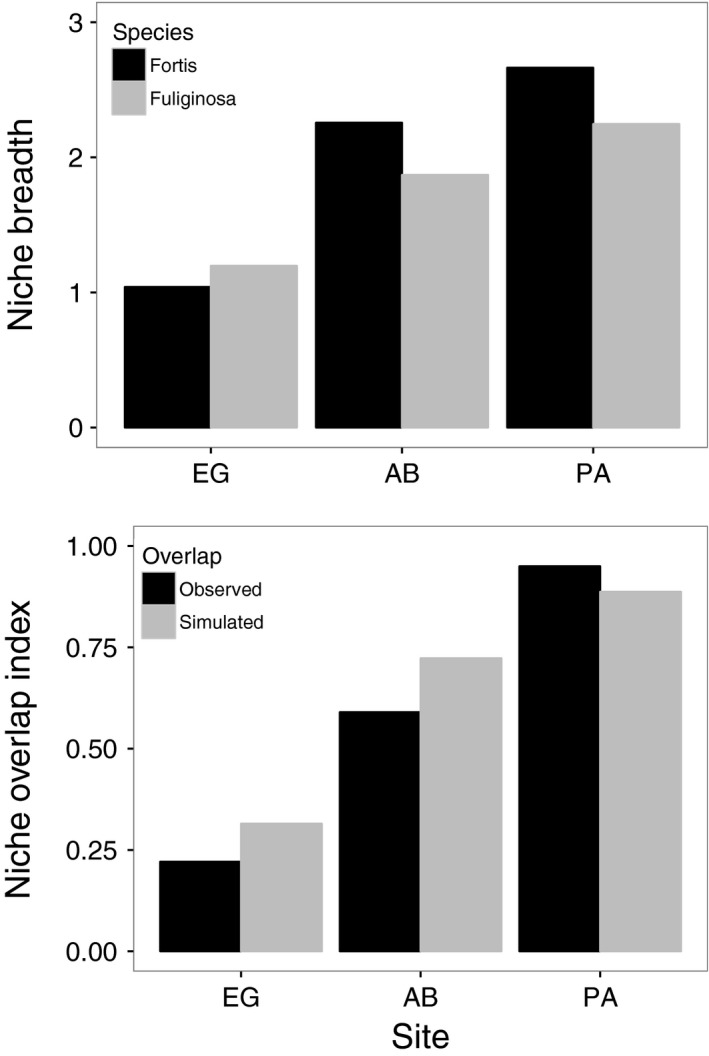
Niche characteristics of ground finches (*Geospiza fortis* and *Geospiza fuliginosa*) across sites with different degrees of urbanization. The data represent the Shannon–Wiener's niche breadth (top panel) and Pianka's niche overlap (bottom panel) index estimated from feeding observations at three sites on Santa Cruz Island, Galápagos, Ecuador. Site labels are El Garrapatero (EG), Academy Bay (AB), and Puerto Ayora (PA)

Across sites, variations in resource use led to highly variable estimates of niche breadth within species and niche overlap between species. In the case of *G. fortis* and *G*. *fuliginosa*, niche breadths were lowest at EG (non‐urban site), intermediate at AB (intermediate urban site), and highest at PA (urban site; Figure 5), consistent with variation in food type diversity. With respect to diet partitioning between these two species, finches at EG (non‐urban site) and AB (intermediate urban site) showed lower diet overlap than expected from random simulations (Figure 5), whereas the two finch species at PA (urban site) showed nearly 100% diet overlap, greater than expected at random (Figure 5). Similar trends emerged in analyses of all ground finch species, both for niche breadth (Supporting Information Figure S3) and for niche overlap (Supporting Information Figure S4).

## 4 DISCUSSION

Our main results were as follows: (a) finch density was notably higher at urban sites than at non‐urban sites; (b) food type availability and the diet of finches at urban sites were notably broader than at other sites and included many human foods; (c) finches at sites frequented by people (the urban sites and the non‐urban tourist site) were willing to approach people and novel objects (food trays), much more so than were finches at the non‐urban site; (d) the degree of urbanization and the presence of humans associate closely with strong preferences for human foods, with finches at urban sites feeding predominantly on human foods. From these findings, we can surmise that finch preferences for human foods in urban environments, and corresponding alterations to their behavior, have at least for the moment collapsed niche differences that normally characterize the adaptive radiation of Darwin's ground finches. These results also suggest that urbanization and the introduction of novel ecological resources are modifying finch adaptive landscapes and behavior, in ways and to degrees that seem likely to undermine the natural processes that drive adaptive diversification.

### 4.1 Behavioral flexibility and adaptation to urban environments

Urbanization often alters the distribution of ecological resources (McKinney, 2002, 2006 ; Schochat, Warren, Faeth, McIntyre, & Hope, 2006) and represents a novel and strong agent of selection to which organisms might or might not adapt (Alberti, 2015; Atwell et al., 2012; Smith & Bernatchez, 2008). One way that organisms could cope with altered resources is through behavioral flexibility, defined as the ability of organisms to alter their behavior in response to changing environments (Coppens, Boer, & Koolhaas, 2010). Behavioral flexibility is expected to facilitate the exploration of novel ecological resources (Inouye, 1978; Sol et al., 2014; Sol, Lefebvre, & Rodríguez‐Teijeiro, 2005; Sol, Timmermans, & Lefebvre, 2002; Tebbich, Sterelny, & Teschke, 2010; Wright et al., 2010), in both natural environments (Liebl & Martin, 2014; Nicolakakis et al., 2003; Sol et al., 2005) and urban environments (Bowers & Breland, 1996; Gotanda, Sharpe, & Léon, 2015; Lowry et al., 2013; Martin & Fitzgerald, 2005; Schochat et al., 2006; Sol et al., 2014). In birds, behavioral flexibility in urban environments is sometimes associated with a reduction in neophobia (Atwell et al., 2012; Boogert, Reader, & Laland, 2006; Martin & Fitzgerald, 2005; Sol et al., 2014) and the incorporation of novel human foods into diets (Boogert et al., 2010; Ducatez et al., 2015; Shochat, Lerman, Katti, & Lewis, 2004; Sol et al., 2002; Sol, Griffin, Bartomeus, & Boyce, 2011; Sol, Lapiedra, & González‐Lagos, 2013).

Consistent with these findings from other systems, we found that Darwin's ground finches in urban environments are accustomed to the presence of people and also display a strong preference for human foods, in contrast to finches from non‐urban environments. In addition*,* the fact that finches at urban sites largely ignored natural foods from experimental trays suggests that behavioral flexibility is facilitating specialization on high‐calorie and easily accessible human foods. For instance, the strong preference for rice at AB (Figure 4) could reflect a canalized/learned behavior, given that finches have previously—on many occasions—eaten rice at this site, furnished by sources such as bird feeders, direct feeding by people, and local restaurants (De León et al., 2011). The current finch preference for human foods could also reflect the influences of both behavioral flexibility and experience operating at different points of time. For example, behavioral flexibility may have been most important as the first generation of urban “pioneers” expanded their diets to include human foods. However, in subsequent generations, urban nestlings raised on human diets may have developed a preference for these foods simply based on experience/familiarity. Nevertheless, we cannot disentangle the relative importance of these two mechanisms with the current data. Overall, however, these findings support the likely importance of behavioral flexibility in promoting Darwin's finch adaptive radiation (Grant & Grant, 2008; Tebbich et al., 2010).

Exploiting urban environments might present additional challenges for organisms (Chamberlain et al., 2009; Lowry et al., 2013; McKinney, 2006; McLaughlin, Janousek, McCarty, & Wolfenbarger, 2014; Slabbekoorn & Peet, 2003), including negative effects on health that might reduce lifespan and probabilities of survival (Salmo, Nilsson, Nord, Bensch, & Isaksson, 2016). In addition, consuming highly processed human foods such as bread and crackers could have negative impacts on finch health or physiological condition (Jones, 2011; Murray et al., 2018), a possibility that should be explored in further studies. Indeed, urban environments could constitute effective ecological traps where organisms exploit environments with negative fitness consequences (Dwernychuk & Boag, 1972). In short, while our results clearly show a shift to human foods in urban sites, the adaptive significance of that shift remains to be determined. Examining the physiological and health effects of consuming human foods seems crucial to understanding the potential fitness and evolutionary consequences of urbanization on Darwin's finches.

### 4.2 The urban finch and the future of adaptive radiation

Accumulating evidence illustrates that some species are able to exploit novel resources provided by urban environments (Donihue & Lambert, 2014; Johnson & Munshi‐South, 2017; Kettlewell, 1955; Littleford‐Colquhoun et al., 2017; Sol et al., 2002; Winchell et al., 2016). Less clear, however, are the evolutionary consequences of using these novel resources for populations or species undergoing adaptive radiation. In Darwin's finches, the interaction between resource availability and competition for resources is thought to be essential for promoting diversification and then maintaining coexistence among closely related species (Bowman, 1961; De León et al., 2014; Grant, 1999; Lack, 1947; Schluter, 2000). Indeed, these processes have led to the formation of a number of species whose beak morphology is differentially adapted to feed on different food resources (Bowman, 1961; Grant, 1999; Lack, 1947; Schluter, 2000). However, Darwin's finches might also be considered opportunistic or “imperfect generalists” (sensu De León et al., 2014) because, during benign periods, their diets tend to converge on foods that are abundant and easily accessible, regardless of their beak morphology, resulting in temporary weakening of selection (Abbott et al., 1977; Smith, Grant, Grant, Abbott, & Abbott, 1978). Nevertheless, strong selection on beak morphology re‐emerges during periods of drought or scarcity, when finches specialize on the food types for which they are best adapted. As a consequence, year‐round availability of soft and highly abundant human foods in urban environments is likely to affect the very ecological and evolutionary processes that promote species and phenotypic diversification in Darwin's finches (De León et al., 2011; Hendry et al., 2006).

As suggested by our results, in the presence of an abundance of calorie‐rich and readily available human foods, natural inter‐species ecological differences might be eroding, leading to smoothing of the previously rugged adaptive landscape and a corresponding weakening of selection underlying divergence. This process has been inferred previously adjacent to urban environments on Santa Cruz Island, where divergent morphs of the medium ground finch have been progressively converging as the human population has increased (De León et al., 2011; Hendry et al., 2006). Our present study shows how the effects of urbanization and human behavior might also extend to other ground finch species. Specifically, besides eroding ecological differences between the small (*G. fuliginosa)* and medium (*G. fortis*) ground finch, we observed a substantial number of cactus finch (*G. scandens*, 11 individuals) and large ground finch (*G. magnirostris*, 55 individuals) feeding on and responding to human foods at the urban site. Furthermore, our finding that finches at a non‐urban but tourist site (EG Beach; Supporting Information Movie S1) also elicited a strong preference for human foods suggests that ecological niches might be more susceptible to human disturbances than previously thought. As such, changes in beak morphology within and across species in urban environments could be shaped by reduced survival disadvantages of intermediate beak‐size birds, including hybrid individuals, which under natural circumstances are unlikely to survive (i.e., because they fall in valleys of low fitness). One remaining question is what the consequences of urbanization and the presence of humans at the local scale might be for the adaptive radiation of Darwin's finches as a whole?

One likely short‐term consequence of urbanized adaptive landscapes will be the convergence of previously distinct species through introgressive hybridization (Rhymer & Simberloff, 1996; Seehausen, Takimoto, Roy, & Jokela, 2008; Taylor et al., 2006). In the ground finches, hybridization is common and has been detected in non‐urban habitats (Chaves et al., 2016; Grant, 1993; Grant & Grant, 1989, 1996 ; Lamichhaney et al., 2015), suggesting a lack of intrinsic genetic incompatibilities among species. During extreme climatic conditions (i.e., high rainfall) on Daphne Major, when natural foods abound, hybridization has led to convergence of species such as the cactus finch and the medium ground finch (Grant & Grant, 2002; Grant, Grant, Markert, Keller, & Petren, 2004), and also for the small and the medium ground finch (Grant & Grant, 2016). In tree finches (*Camarhynchus* spp.), hybridization has also been detected on Floreana Island, likely associated with the introduction of the *Philornis* parasite (Kleindorfer et al., 2014; Peters, Myers, Dudaniec, O'Connor, & Kleindorfer, 2017). Overall, these studies suggest that introgressive hybridization in Darwin's finches is widespread. If it is also strong and persistent, it could lead to the collapse of species boundaries in human‐altered environments. Another potential consequence of hybridization in urban environments is the generation of novel genetic variation that could facilitate further diversification in Darwin's fiches. This could occur, for instance, if novel hybrid individuals with intermediate beak sizes are able to experience high fitness by specializing on food items of intermediate size/hardness, such as human foods. It is important to mention that despite evidence of hybridization, other axes of divergence such as differences in song types and vocal performance (Grant, 1999; Huber & Podos, 2006; Podos, 2001) could also help maintain species boundaries in the face of increasing ecological disturbance. However, both song types and vocal performance are tightly associated with beak morphology (Podos, 2001), suggesting that changes in selection pressure on beaks via alteration of food resources could also affect other axes of divergence.

Alteration of ecological resources at local scales such as a single urban site on Santa Cruz Island could potentially have broader implications for the ground finch radiation across the entire island. For instance, increasing finch population at the urban site could promote merging of species via gene flow and interspecific hybridization (as above). This possibility was hinted at by our previous study that showed that genetic differences among ground finches (*G. fortis*,* G. fuliginosa,* and *G. magnirostris*) are smaller at AB (the intermediate‐urban site) than at EG (the non‐urban site) (De León et al., 2010), possibly due to higher gene flow among species at the intermediate‐urban site. Interestingly, *G. fortis* and *G. fuliginosa* are both the most abundant and the most closely related species across sites (Chaves et al., 2016; De León et al., 2010), which is likely to amplify the merging effect of hybridization in urban environments. In addition, urban finch populations could be a source of maladaptive gene flow (Hendry, Taylor, & McPhail, 2002), leading to changes in the optimal beak‐size distribution of non‐urban finch populations. We refer to maladaptive traits as those that reduce fitness under a given environmental condition. For instance, urban finches could ultimately evolve an “urban beak morphology” (e.g., a small and soft bill) adapted to exploit soft human foods in urban environments. But individuals with that morphology would face a lower fitness if they migrated to natural environments where seeds are larger and harder than human foods. In this context, hybridization (or gene flow) from urban environments could render non‐urban finch populations unable to cope with drastic environmental changes under natural conditions and could reinforce genetic differences between urban and non‐urban populations. Thus, we postulate that maladaptation could be another unintended consequence of urbanization.

### 4.3 Possible evolutionary consequences of human behavior

Studies of urbanization often highlight human population density, the presence of impervious surfaces or the development of infrastructure as main drivers of effects on local biodiversity (Alberti, 2015; Gaston, 2010; Gotanda et al., 2017; Johnson & Munshi‐South, 2017; McKinney, 2006). However, human behavior and the way we interact with local biodiversity could expand impacts of urbanization beyond city centers. This was suggested by our finding of strong finch preferences for human foods at EG Beach, a non‐urban but tourist site located ~12 km away from the town of PA. This also suggests that human behavior rather than human population density is the main driver of finch preference for human foods. Although additional replication is needed to statistically disentangle these factors, we argue the effect of human behavior on finch diets is likely facilitated by our tendency to feed birds either directly (via feeders) or inadvertently (via food dropping or littering), both of which are often seen at both urban centers and non‐urban tourist sites (L F. De León, per. obs). In addition, although the Galápagos are a protected area, and human infrastructure is rather localized, popular tourist sites outside the urban areas appear to also be affected by the way human interact with finches at those sites.

The ecological and conservation implications of wildlife feeding and food provisioning have been explored extensively across a diversity of taxa (Cox & Gaston, 2018; Dubois & Cheptou, 2017; Murray et al., 2018). However, less is known about the evolutionary implications of such common human behaviors. Here, we showed that wildlife feeding and food provisioning in Darwin's finches could impact the very evolutionary process the drive diversification in this iconic birds.

In conclusion, our study focused on single urban center on a single island, and the lack of replication limits our ability to draw general inferences. Yet, our study represents a first attempt to explore the potential impacts of urbanization and human behavior on the ongoing adaptive radiation of Darwin's finches. We also hypothesize that a similar phenomenon might currently be affecting finches in urban environments across the Galápagos archipelago—a possibility that we are exploring currently in the coastal and agricultural zones of other islands. Moreover, urban effects on finch evolution might be comparatively strong, given their persistence (continued access to human foods) as opposed to the episodic nature of extreme climatic events. Our study also adds to the increasing evidence of human effects on Galápagos biodiversity. This includes the near‐extinction of the Mangrove finch resulting from the introduction of a parasitic fly (Fessl et al., 2010; Fessl, Dvorak, Vargas, & Young, 2011), and the collapse of multiple unique plant and animal species due to habitat modification, and the introduction and proliferation of alien species (Mauchamp, 1997; Rentería, Gardener, Panetta, Atkinson, & Crawley, 2012; Trueman, Atkinson, Guézou, & Wurm, 2010; Watson, Trueman, Tufet, Henderson, & Atkinson, 2010).Our findings thus accentuate the fragility of the initial stages of adaptive radiation in Darwin's finches and raise concerns about the fate of the Galápagos ecosystems in the face of increasing urbanization and human presence. Ultimately, understanding the unexpected consequences of urbanization on ecological niches might guide strategies for preserving biodiversity and the processes that generate it.

## CONFLICT OF INTEREST

None declared.

## Data Availability

Data are available in the Supporting Information.
