# Supplementary material for: Urbanization erodes niche segregation in Darwin's finches
Source: Evol Appl. 2018 Dec 18;12(7):1329–43. doi: 10.1111/eva.12721 (PMC6691225; doi:10.1111/eva.12721)
Supplement: Supplementary file 1 [file EVA-12-1329-s001.docx]

**Supplementary information**


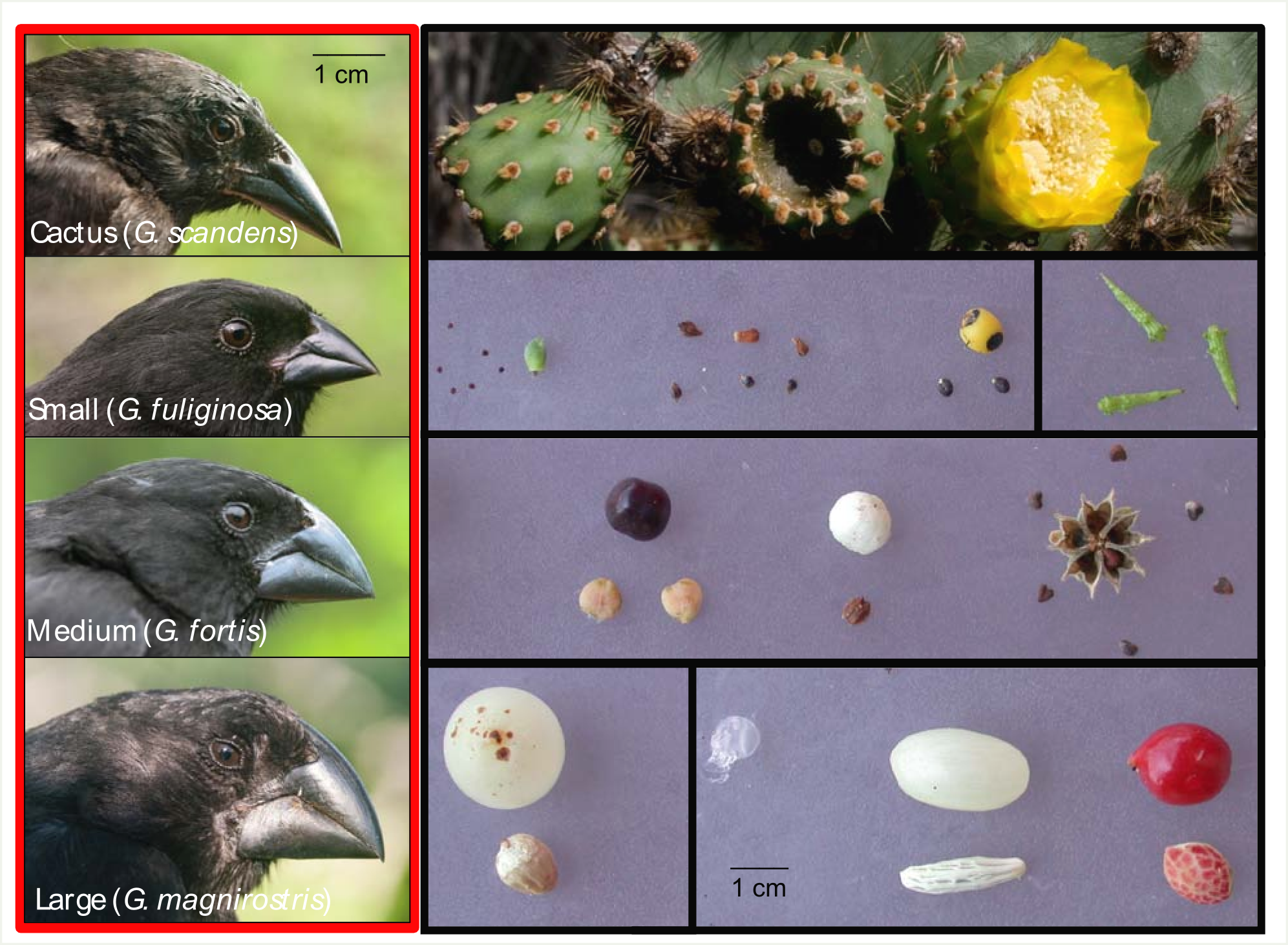


**Fig. S1**. Darwin’s ground finches and some of their natural foods. Four species are shown from top to bottom. The cactus finch (*Geospiza scandens*) with flowers and fruit of the cactus *Opuntia echios.* The small ground finch (*G. fuliginosa*) with fruit and seeds of *Portulaca oleracea*, C*ryptocarpus pyriformes* and *Tournefortia psilostachya*, and fruits of *Commicarpus tuberosus*. The medium ground finch (*G. fortis*) with fruits and seeds of *Scutia spicata* and *Tournefortia pubescens*, and a *Bastardia viscosa* seed case and seeds. The larger ground finch (*G. magnirostris*) with a fruit and seed of *Cordia lutea*, *Vallesia glabra*, and *Castela galapageia* (Photo credit: L. F. De León).

**
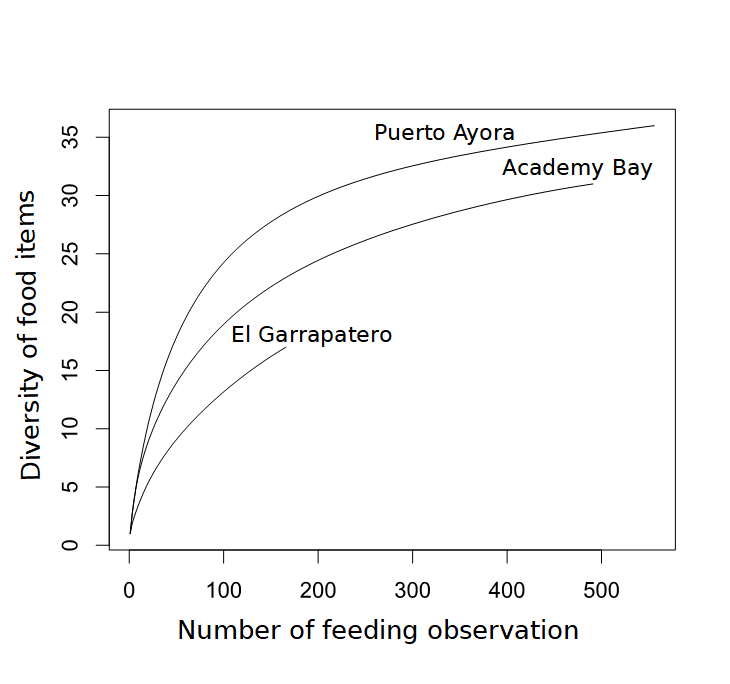
**

**Fig. S2.** Rarefaction curves describing the accumulation of human food items as a function of the number of feeding observation at each sampling site on Santa Cruz Island, Galápagos, Ecuador.


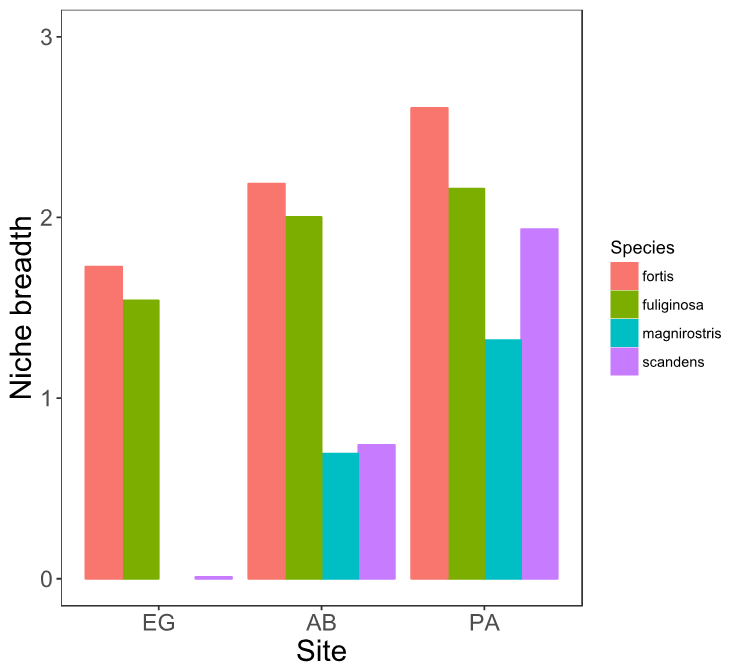


**Fig. S3.** Niche breadth for coexisting species of ground finches across sites with different degrees of urbanization. The data represent Shannon-Wiener indices estimated from feeding observations at three sites on Santa Cruz Island, Galápagos, Ecuador. The four species are present at the three sites, but no feeding observations were recorded for *G. manirostris* at EG. Site labels represent El Garrapatero (EG), Academy Bay (AB), Puerto Ayora (PA).


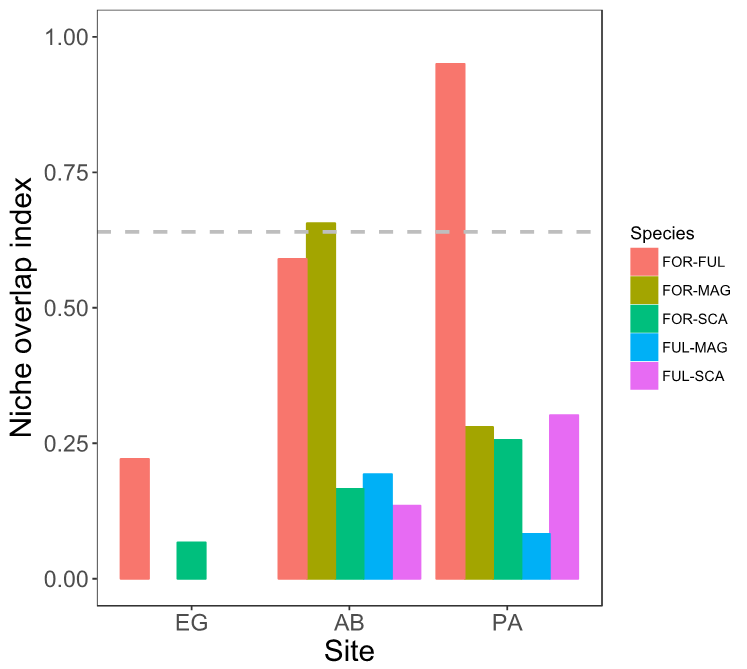


**Fig. S4.** Pairwise niche overlap in four coexisting species of ground finches across sites with different degrees of urbanization. The data represent Pianka’s (1973) overlap indices estimated from feeding observations at three sites on Santa Cruz Island, Galápagos, Ecuador. Dashed line show the expected niche overlap estimated from null models. The four species are present at the three sites, but no feeding observations were recorded for *G. manirostris* at EG. Site labels represent El Garrapatero (EG), Academy Bay (AB), Puerto Ayora (PA).

**Table S1.** Total number of food items recorded in the diet of Darwin's finches and other common bird species at different sites on Santa Cruz Island, Galápagos, Ecuador.

| Foods | Origin | El Garrapatero | Academy Bay | Puerto Ayora | Total |
| --- | --- | --- | --- | --- | --- |
| *Acacia sp.* | Natural | 2 | 4 | 1 | 7 |
| *Alternalthera sp.* | Natural |  |  | 1 | 1 |
| Beans | Human |  |  | 16 | 16 |
| *Boerhavia caribaea* | Natural | 4 | 11 | 14 | 29 |
| Bread | Human |  | 2 | 29 | 31 |
| *Bursera graveolens* | Natural |  | 2 |  | 2 |
| Candy | Human | 2 |  |  | 2 |
| Cantaloupe | Human |  |  | 11 | 11 |
| *Castela galapageia* | Natural |  | 7 |  | 7 |
| Centipede | Natural |  | 2 |  | 2 |
| *Commicarpus tuberosus* | Natural | 1 | 7 | 3 | 11 |
| *Conocarpus erectus* | Natural |  | 8 | 3 | 11 |
| *Cordia leucophlyctis* | Natural |  | 4 | 5 | 9 |
| *Cordia lutea* | Natural | 1 | 32 | 13 | 46 |
| Corn | Human |  |  | 38 | 38 |
| Cracker | Human |  |  | 16 | 16 |
| Unidentified crumbs | Human | 1 | 6 | 205 | 212 |
| *Cryptocarpus pyriformis* | Natural | 9 | 78 | 7 | 94 |
| *Delonix regia* | Introduced |  | 1 | 46 | 47 |
| Grass (several species) | Natural/Introduced |  |  | 4 | 4 |
| Ground | Natural | 117 | 114 | 20 | 251 |
| *Heliotropium sp.* | Natural |  | 1 |  | 1 |
| Hibiscus | Introduced |  | 1 | 7 | 8 |
| Insect | Natural | 1 | 7 | 7 | 15 |
| *Laguncularia racemosa* | Natural |  | 1 |  | 1 |
| *Lantana peduncularis* | Natural |  |  | 1 | 1 |
| Lichen | Natural |  | 2 |  | 2 |
| *Maytenus* octogona | Natural |  | 1 |  | 1 |
| *Ocimum basilicum* | Introduced |  |  | 1 | 1 |
| *Opuntia echios* | Natural | 6 | 38 | 4 | 48 |
| Orange | Human |  |  | 1 | 1 |
| *Parkinsonia aculeata* | Natural | 1 | 2 |  | 3 |
| *Passiflora foetida* | Natural |  |  | 16 | 16 |
| Pastry | Human |  |  | 3 | 3 |
| Fried plantain | Human |  |  | 8 | 8 |
| *Portulaca oleracea* | Natural |  | 8 | 7 | 15 |
| *Prosopis sp.* | Natural | 2 |  |  | 2 |
| Rice | Human |  |  | 25 | 25 |
| *Scutia spicata* | Natural | 14 | 104 | 7 | 125 |
| *Sesuvium portulacastrum* | Natural |  | 9 | 5 | 14 |
| *Sida sp.* | Natural |  | 1 |  | 1 |
| Spondias | Human |  |  | 2 | 2 |
| Tiquila | Natural | 1 |  |  | 1 |
| *Tournefortia psilostachya* | Natural | 1 | 5 | 6 | 12 |
| *Tournefortia pubescens* | Natural |  | 2 |  | 2 |
| *Tribulus* | Natural |  | 2 | 5 | 7 |
| *Vallesia glabra* | Natural | 2 | 5 |  | 7 |
| Waffle | Human |  |  | 1 | 1 |
| *Waltheria ovata* | Natural | 1 |  |  | 1 |
| Water | Human |  | 24 | 11 | 35 |
| Watermelon | Human |  |  | 7 | 7 |
| Total |  | 166 | 491 | 556 | 1213 |

**Table S2**. Abundance of birds observed during 75 hours of survey across sites on Santa Cruz Island, Galápagos, Ecuador.

| Species | Common name | Academy Bay | EG beach | El Garrapatero | Puerto Ayora | Total |
| --- | --- | --- | --- | --- | --- | --- |
| *Crotophaga ani* | Smooth-billed ani | 19 | 9 | 10 | 36 | 74 |
| *Camarhynchus parvulus* | Small tree finch | 147 | 3 | 4 | 50 | 204 |
| *Dendroica petechia* | Yellow warbler | 85 | 5 | 2 | 67 | 159 |
| *Geospiza fortis* | Medium ground finch | 363 | 70 | 145 | 594 | 1172 |
| *Geospiza fuliginosa* | Small ground finch | 194 | 35 | 39 | 289 | 557 |
| *Geospiza* spp. | Ground finch | 278 | 77 | 190 | 360 | 905 |
| *Geospiza magnirostris* | Large ground finch | 9 |  | 1 | 9 | 19 |
| *Mimus parvulus* | Galápagos mockingbird | 95 | 31 | 42 | 55 | 223 |
| *Myiarchus magnirostris* | Galápagos flycatcher | 59 | 11 | 9 | 23 | 102 |
| *Platyspiza crassirostris* | Vegetarian finch | 12 |  | 3 | 17 | 32 |
| *Geospiza scandens* | Cactus finch | 67 | 1 | 13 | 57 | 138 |
| Total | Total | 1328 | 242 | 458 | 1557 | 3585 |

**Table S3**. Finch response to human foods across sites on Santa Cruz Island, Galápagos Ecuador. The values represent post-hoc Dunn tests for multiple comparisons (t-statistics and p-values in brackets) for the number of finches approaching the food trays (upper half) and the human subject (lower half) during feeding experiments. Values in bold represent statistically significant differences after Bonferroni corrections.

|  | El Garrapatero | EG Beach | Academy Bay | Puerto Ayora |
| --- | --- | --- | --- | --- |
| El Garrapatero |  | 6.69 (<**0.001**) | 0.47 (1.000) | -3.95 (<**0.001**) |
| EG Beach | -7.23 (<**0.001**) |  | -5.76 (<**0.001**) | 2.89 (**0.011**) |
| Academy Bay | -1.25 (0.635) | -4.85 (<**0.001**) |  |  |
| Puerto Ayora | -2.96 (**0.009**) | -4.54 (0.634) | -1.25 (0.634) |  |

**Video 1.** Finch response to human foods at EG Beach. Video credit: D. Sharpe

**Video 2.** Finch response to human foods at Puerto Ayora. Video credit: K. Gotanda.
